# Effectiveness of Probiotic Lozenges in Periodontal Management of Chronic Periodontitis Patients: Clinical and Immunological Study

**DOI:** 10.1055/s-0040-1709924

**Published:** 2020-05-21

**Authors:** Ahmed Alshareef, Alaa Attia, Mohammed Almalki, Faisal Alsharif, Ahmed Melibari, Bassel Mirdad, Ehab Azab, Abdel-Rahman Youssef, Ahmed Dardir

**Affiliations:** 1 Faculty of Dentistry, Umm Al-Qura University, Makkah, Saudi Arabia; 2 Faculty of Dentistry, Al-Azhar University, Assiut, Egypt; 3 Department of Microbiology, Faculty of Medicine, Suez Canal University, Ismailia, Egypt; 4 Faculty of Dentistry, Al-Ahram Canadian University, Giza Governorate, Egypt

**Keywords:** probiotic lozenges, GCF/MMP-8, chronic periodontitis, periodontal therapy

## Abstract

**Objective** The main purpose of this article was to evaluate the effect of probiotics used as an adjunctive to scaling and root planing (SRP) on the periodontal parameters and matrix metalloproteinase-8 (MMP-8) levels in gingival crevicular fluid (GCF) of chronic periodontitis patients.

**Materials and Methods** A total of 25 chronic periodontitis patients who completed the treatment course of 40 subjects, aged 25 to 58 years, participated in this study. They were categorized into two groups: the first group was treated by SRP while the second group was treated by SRP and probiotic lozenges twice a day for 30 days. All patients were evaluated clinically by measuring the plaque index, bleeding index (BI), pocket depth, clinical attachment loss, and immunologically by assaying GCF/MMP-8 at baseline and 30 days after periodontal management.

**Results** There was a significant improvement in periodontal parameters after SRP treatment with and without probiotic lozenges in both groups. However, there was a significant decrease in the BI ( *p* = 0.05) in SRP and probiotic lozenges group after 30 days compared with SRP alone. In addition, there was a significant decrease in GCF/MMP-8 levels after 30 days in patients managed by SRP only ( *p* = 0.017) compared with the baseline in both groups, whereas a highly significant decrease in patients treated by SRP and probiotics ( *p* = 0.001).

**Conclusion** The current study suggested that the probiotics might have a beneficial effect on clinical and immunological outcomes in the management of chronic periodontitis patients. Further research is needed on a large-scale population and for a long recall time to confirm the response to probiotics as an adjunctive to SRP.

## Introduction

Periodontitis is an infection caused by specific microorganisms that produce inflammation and destruction of periodontium. The chronic periodontitis is the most common type of periodontal disease that results in periodontal defects, intermittent pain, and, finally, losing the tooth. The risk factors of periodontitis include a combination of environmental, acquired and genetic factors. Therefore, microbial plaque and host response are the critical factors for the developing of chronic periodontitis. 1

Generalized periodontitis is considered when 30% of the sites assessed in the mouth show bone and attachment loss, while localized periodontitis when < 30% of the evaluated sites are affected. 2 The rate of the disease activity is usually slow but may be modified by systemic, local, and behavioral factors. Chronic periodontitis occurs mainly in adult and old-age patients in association with local factors, primarily dental plaque. The severity of periodontitis is different from patient to patient and from site to site in the same patient. 3

Chronic periodontitis is treated by both nonsurgical and surgical approaches. Nonsurgical periodontal therapy includes oral hygiene instructions, patient education, scaling and root planing (SRP), and occlusal adjustment, if needed. A patient who is diagnosed with periodontitis in the early stage will achieve a better prognosis. However, teeth with residual deep pockets and irregular gingival and bony margins will be treated surgically, either through resective, regenerative, or combined approaches. 4 The effectiveness of periodontal therapy is made possible by the remarkable healing capacity of the periodontal tissues. 5 Periodontal treatment can reduce the pain, gingival changes, bleeding, and pocket depth (PD), prevent soft tissue and bone destruction, and decrease tooth mobility. 6

Besides nonsurgical periodontal therapy (SRP), there are several adjunctive modalities used to improve periodontal outcomes such as local and systemic antibiotics, antimicrobial agents, photodynamic therapy, and the use of probiotics. 7 Probiotics are live microorganisms that provide health benefits when ingested by improving or restoring the gut flora. Probiotics repopulate the good bacteria, which can help to fight pathogenic organisms and resist against infection. Administration of probiotics as an adjunctive besides the scaling may improve oral health conditions either by inhibiting the growth of the pathogenic microorganisms or by modifying the mucosal immunity in the oral tissues. 8 Health benefits have been shown with a specific strain of probiotics of the following species: *Lactobacillus* , *Leuconostoc* , *Bacillus* , *Escherichia coli* , *Bifidobacterium* , *Saccharomyces* , *Enterococcus* , *Streptococcus* , ** and *Pediococcus* . 9

*Viridans streptococci* are commensal, facultative anaerobic, Gram-positive bacteria that form significant part of the normal flora of the human oral cavity. *Lactobacillus* is a Gram-positive aero-tolerant but prefers to grow in an anaerobic atmosphere. 10 *Lactobacillus* is more significant in the progression of caries cavitation than the invasion of the periodontium. 11 When using a single strain or a combination of different strains, the probiotics provide beneficial effects to the patient depending on the patient’s response. 12 Therefore, probiotics might affect the periodontium when used as an adjunctive to periodontal therapy. In a previous study, the authors observed that when probiotic intake was stopped, the percentage of sites that bleed upon probing increased again. 13

Probiotics are a promising area of research, especially in the management of the periodontal disease. *Streptococcus oralis* and *Streptococcus uberis* have reported delaying growth of pathogenic microorganisms in laboratory and animal models. They are indicators of healthy periodontal tissue. When the number of healthy organisms is decreased, the number of pathogenic microorganisms is increased. 14 Probiotics utilize naturally occurring microorganisms to confer health benefits when given in adequate amounts. It refers to genera of bacteria capable of halting, altering, or delaying periodontitis. It possesses a proper scope in the field of periodontitis. It can be used to reduce plaque accumulation, modify anaerobic microorganism colonization, reduce PD, improve the clinical attachment level, and prevent recolonization of pathogenic bacteria. 15

The destruction of the periodontium is mainly due to enzymes released during the inflammatory process, 16 notably the matrix metalloproteinases (MMPs). The MMPs are group of subjective enzymes that plays a significant effective role in both physiological and pathological mechanisms. 17 Matrix metalloproteinase-8 (MMP-8) is one of the MMPs that is present in gingival crevicular fluid (GCF) of periodontitis patients, and it may be an indicator of the presence of periodontal inflammation. 18 High levels of MMP-8 in GCF and saliva, 19 as well as the increase in the expression of MMP-8 in gingival tissue, were identified in periodontitis patients. 20 Nonsmoking untreated chronic periodontitis subjects expressed higher levels of MMP-8 in the periodontium than healthy periodontal subjects. 21

The knowledge regarding the benefits of probiotics in the treatment of chronic periodontitis is limited. The aim of this study was to evaluate the effects of probiotics on the periodontal parameters and MMP-8 levels in GCF of chronic periodontitis patients before and after nonsurgical periodontal therapy with and without the adjunctive use of probiotics.

## Materials and Methods

### Subjects

The study was designed as a randomized clinical trial and the proposal was approved by the Institutional Review Board of Umm Al-Qura University. The participants of this study were recruited from Umm Al-Qura University’s dental hospital and provided a written informed consent. Twenty five individuals aged between 25 and 58 years with mean age 29 ± 96 years completed the treatment course of 40 subjects at baseline of periodontal management. The inclusion criteria of selected individual were systemically healthy, and were having moderate to severe chronic periodontitis (CAL ≥ 3 mm). Patients with the following criteria were excluded from this study: having pregnancy, receiving any form of periodontal therapy in the past 6 months, using analgesics regularly, and used antibiotics in previous 3 months. The study was performed on 25 individuals with moderate to severe chronic periodontitis. The 25 participants were categorized into two groups. The first group ( *N* = 10) received SRP only, and the second group ( *N* = 15) received SRP and probiotic lozenges, and both groups were evaluated after 30 days from the periodontal therapy.

### Prescription of Probiotic Lozenges

The probiotic lozenges (Flora, Inc., Canada) were given twice a day to the second group after SRP. Each probiotic lozenge contains five bifid bacteria including *Lactobacillus acidophilus* , *Lactobacillus casei* , *Bifidobacterium bifidum* , *Lactobacillus rhamnosus* , ** and *Lactobacillus salivarius* .

### Clinical Examination and Periodontal Therapy

The periodontal parameters included simplified plaque index (PI), 22 bleeding index (BI), 23 pocket depth (PD), and clinical attachment loss (CAL), 24 and were measured for all individuals in both groups before and after periodontal treatment. All participants received nonsurgical periodontal therapy including SRP, oral hygiene instructions, polishing, and chemical washing. The SRP was performed on two alternative visits to insure removing of supra and subgingival calculus, meticulous root planning, and management of deep pockets by using ultrasonic scaler and hand instruments (scalers and Gracey curates). All individuals were instructed to comply with oral hygiene measures to keep plaque in control through the course of evaluation. Both groups were evaluated after 30 days to measure the clinical parameters and to obtain GCF samples.

### GCF Samples and Assaying of MMP-8

Before GCF collection, all the supragingival deposits were removed gently without any trauma to the gingival crevice. Thereafter, 3 µL of GCF were collected using micropipette capillary tubes from the pockets of right and left maxillary quadrant, and then the area was isolated to avoid salivary contamination. The GCF samples were collected from all the patients at baseline and after 30 days of periodontal management. The GCF sample was diluted in 200 µL of phosphate buffered solution in 1.5 mL Eppendorf tube, then stored at–80°C until use. The levels of MMP-8 were measured using human MMP-8 ELISA kit (Abcam, UK) according to manufacturer instructions. Briefly, 50 µL of standards or samples were added to appropriate wells. Then, a 50 µL antibody cocktail (capture and detector antibodies) was added to all wells and preserved at room temperature for 1 hour. After washing three times, we added 100 µL of 3,3′,5,5′-Tetramethylbenzidine (TMB) Liquid Substrate to each well and incubated for 10 minutes. Then the reaction was stopped by using 100 µL stop solution and the optical density values were read at 450 nm using SPECTRO star Nano BMG LABTECH ELISA reader.

### Statistical Analysis

The collected data were analyzed by using Statistical Package for the Social Sciences (SPSS) version 22 software program. The descriptive analysis (minimum, maximum, range, mean, and standard deviation [SD]) was included in the software. The comparison before and after treatment for the same group was made by a paired *t* -test, while the comparison between groups was made by the unpaired student’s *t* -test. Statistically significant difference was obtained at *p* ≤0.05. The data were tabulated and displayed in figures using the Microsoft Word program (version 13).

## Results

### Evaluation of Periodontal Parameters

**Fig. 1:**
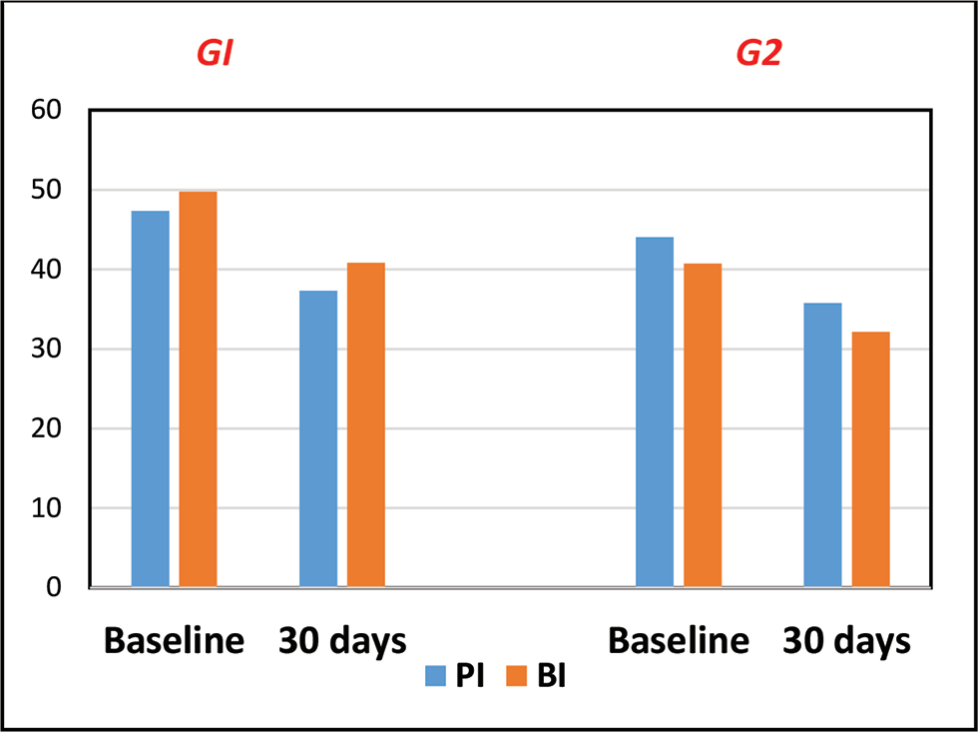
Demonstration of the mean values of plaque (PI) and bleeding index (BI) in both examined groups.

The descriptive statistics of the periodontal parameters for both groups were expressed as mean, SD, and *t* - and *p* -values ( Tables 1 2 ; Figs. 1 2 ). At the baselines, the mean of periodontal parameters for Group 1 was PI = 47.32% (SD =15.38), BI = 49.75% (SD = 13.93), PD = 2.61 mm (SD = 0.41), and CAL = 3.49 mm (SD = 0.66), while for Group 2, the mean was PI = 44.01% (SD = 16.06), BI = 40.75% (SD = 9.58), PD = 2.55 mm (SD = 0.23), and CAL = 3.57 mm (SD = 0.58). Thirty days after periodontal treatment with or without probiotic lozenge, the mean of periodontal parameters of Group 1 was PI = 37.31% (SD = 12.29), BI = 40.82% (SD = 13.21), PD = 2.3 mm (SD = 0.43), and CAL = 3.14 mm (SD = 0.65), while for Group 2, the mean was PI = 35.74% (SD = 15.18), BI = 32.15% (SD = 8.5), PD = 2.19 mm (SD = 0.27), and CAL = 3.14 mm (SD = 0.59).

**Table 1:** Statistical comparisons of clinical periodontal parameters for both examined groups

	Parameters	Compression	Mean	SD	*t* -test	*p* -Value
G1	PI	Baseline	47.3250	15.38717	3.945	0.003*
		30 d	37.3160	12.29990		
	BI	Baseline	49.7550	13.93193	6.974	0.000*
		30 d	40.8200	13.21242		
	PD	Baseline	2.6130	0.41508	5.234	0.001*
		30 d	2.3380	0.43235		
	CAL	Baseline	3.4930	0.66101	8.557	0.000*
		30 d	3.1490	0.65514		
G2	PI	Baseline	44.0127	16.06966	5.635	0.000*
		30 d	35.7433	15.18255		
	BI	Baseline	40.7533	9.58256	4.843	0.000*
		30 d	32.1533	8.50200		
	PD	Baseline	2.5533	0.23654	7.744	0.000*
		30 d	2.1973	0.27830		
	CAL	Baseline	3.5740	0.58024	4.932	0.000*
		30 d	3.1487	0.59010		
Abbreviations: BI, bleeding index; CAL, clinical attachment loss; d, days; PD, pocket depth; PI, plaque index; SD, standard deviation. Note: G1 = patients treated without probiotics; G2 = patients treated with probiotics. *The p-value < 0.01 (was considered highly significant).						

**Table 2:** Unpaired t-test statistical comparisons of clinical parameters for both examined groups

	Parameters	Compression	Mean	SD	*t* -Test	*p* -Value
G1	PI	G1 (baseline)	47.3250	15.38717	0.513	0.613
		G2 (baseline)	44.0127	16.06966		
	BI	G1 (baseline)	49.7550	13.93193	1.920	0.067
		G2 (baseline)	40.7533	9.58256		
	PD	G1 (baseline)	2.6130	0.41508	0.459	0.651
		G2 (baseline)	2.5533	0.23654		
	CAL	G1 (baseline)	3.4930	0.66101	–0.324	0.749
		G2 (baseline)	3.5740	0.58024		
G2	PI	G1 (30 d)	37.3160	12.29990	0.273	0.787
		G2 (30 d)	35.7433	15.18255		
	BI	G1 (30 d)	40.8200	13.21242	2.003	0.05*
		G2 (30 d)	32.1533	8.50200		
	PD	G1 (30 d)	2.3380	0.43235	0.993	0.331
		G2 (30 d)	2.1973	0.27830		
	CAL	G1 (30 d)	3.1490	0.65514	0.001	0.999
		G2 (30 d)	3.1487	0.59010		
Abbreviations: BI, bleeding index; CAL, clinical attachment loss; d, days; PD, pocket depth; PI, plaque index; SD, standard deviation. Note: G1 = patients treated without probiotics; G2 = patients treated with probiotics. *The p- value < 0.05 (was considered significant).						

**Fig. 2:**
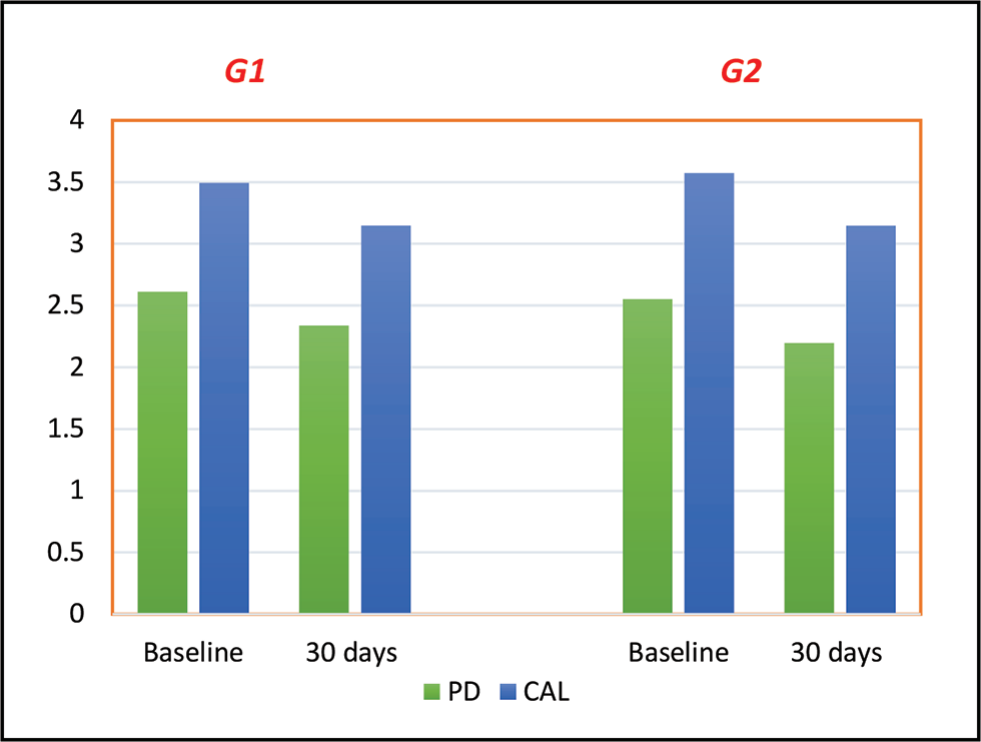
Demonstration of the mean values of pocket depth (PD) and clinical attachment loss (CAL) in both examined groups.

The statistical analysis demonstrated a significant decrease in the periodontal parameters after the nonsurgical periodontal treatment with or without probiotic lozenge, which were as follows for Group 1: PI, *p* = 0.003; BI, *p* = 0.000; PD, *p* = 0.001; and CAL, *p* = 0.000. The parameter values for Group 2 were: PI, *p* = 0.000; BI, *p* = 0.000 ( Fig. 1 ); PD, *p* = 0.000; and CAL, *p* = 0.000 ( Table 1 Figs. 1 2 ). There was no statistically significant difference between Group 1 and Group 2 at 30 days after nonsurgical periodontal treatment with or without probiotic lozenges (PI, *p* = 0.787; PD, *p* = 0.331; and CAL, *p* = 0.999), except with a significant decrease in the BI ( *p* = 0.05) after 30 days of nonsurgical periodontal treatment with probiotic lozenges compared with the nonsurgical periodontal treatment only ( Table 2 Figs. 1 2 ).

### Assaying of MMP-8 Levels

**Fig. 3:**
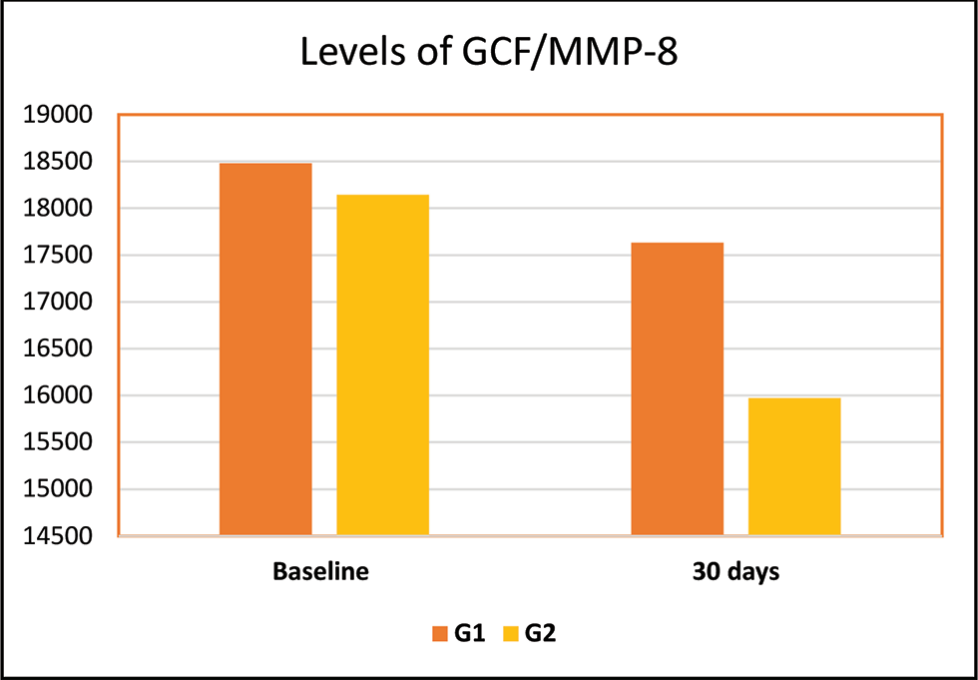
Demonstration of the mean values of gingival crevicular fluid/matrix metalloproteinase-8 (GCF/MMP-8) levels (ng/mL) in both investigated groups.

Descriptive analyses of GCF/MMP-8 levels in Group 1 and Group 2 are summarized in Table 3 . There was a statistically significant decrease in GCF/MMP-8 levels 30 days after periodontal treatment in patients managed by nonsurgical periodontal therapy only compared with the baseline ( *p* = 0.017), whereas there was a highly statistically significant decrease in patients treated by nonsurgical periodontal treatment and probiotic lozenges ( *p* = 0.001). Besides, there were no statistically significant differences in the GCF/MMP-8 levels among Group 1 and Group 2 at baseline ( *p* = 0.524) and 30 days after nonsurgical periodontal therapy ( *p* = 0.257 [ Table 3 ; Fig. 3 ]).

**Table 3:** t-Test statistical comparisons of GCF-MMP8 levels (ng/mL) before and after treatment in both studied groups

	Groups	Comparison	Mean	SD	*t* -Value	*p* -Value
Paired *t* -test	G1	Baseline	18,480.0344	1,119.87508	2.915	0.017*
		30 d	17,632.7918	1,563.30165		
	G2	Baseline	18,144.0245	1,148.91192	3.947	0.001**
		30 d	15,971.9645	2,110.88682		
Unpaired *t* -test	G1	Baseline	18,480.0344	1,119.87508	0.723	0.524
	G2	Baseline	18,144.0245	1,148.91192		
	G1	30 d	17,632.7918	1,563.30165	2.124	0.257
	G2	30 d	15,971.9645	2,110.88682		
Abbreviations: d, days; GCF-MMP8, gingival crevicular fluid-matrix metalloproteinase-8; SD, standard deviation. Note: G1 = patients treated without probiotics; G2 = patients treated with probiotics. *The p- value < 0.05 (was considered significant). **The p-value < 0.01 (was considered highly significant).						

## Discussion

Periodontitis is considered as a multifactorial disease that involves soft and hard periodontal tissues, bacterial colonization, inflammation, and adaptive immune reactions. 25 The treatment modalities of periodontal disease include nonsurgical and surgical management, which emphasizes mostly on mechanical debridement, and, in some cases, are associated with antibiotics. These treatment modalities aimed to eliminate the entire periodontal pathogens that localized and invaded the periodontal tissues. Because of the development resistance of antibiotic and recurrent recolonization of managed sites with pathogenic organisms, there was a need for a new therapy model that can be introduced to periodontal disease. 26

Probiotics are multiple strains of useful bacteria that, when consumed adequately, provide many health benefits to the host. Several actions of probiotics have been proposed in the literature, including killing or inhibition of pathogenic bacterial growth, modification of host immunity by reduction of pro-inflammatory and elevation of anti-inflammatory cytokines, and alteration of cell proliferation and apoptosis. 27 28 Regarding previous knowledge of probiotics as an adjunctive therapy, recently introduced in the field of periodontics, the current study aimed to evaluate the impact of probiotics as an adjunctive treatment to nonsurgical periodontal treatments on the clinical periodontal outcomes as well as on the GCF level of MMP-8 in chronic periodontitis patients.

There are several clinical studies that investigated the clinical efficiency of probiotics in the management of periodontitis in addition to nonsurgical periodontal therapy. 15 29 30 31 32 A systematic review study by Martin-Cabezas et al supported the adjunctive use of *Lactobacillus reuteri* with SRP in the management of chronic periodontitis. 33

The concept of our study evaluated the immunological response and clinical outcomes to periodontal therapy with and without the use of probiotics; this study was supported by previous studies, 31 34 which investigated the effects of probiotics as an adjunctive to nonsurgical periodontal therapy on the levels of some inflammatory cytokines.

The present study showed highly significant improvements in all clinical parameters (PI, BI, PD, CAL) 30 days after SRP treatment with or without probiotic lozenges in both groups. These findings are in agreement with numerous studies. 8 15 30 32 However, intergroup comparison between chronic periodontitis patients with 30 days of nonsurgical periodontal therapy revealed nonsignificant improvements in the clinical parameters. These observations are conflicting with some previous publications. 15 31 For clarification, the nonsignificant improvements in clinical parameters (PI, PD, and CAL) may be due to a short evaluation time of the study.

The statistical comparison of BI in both groups at 30 days showed a significant decrease in patients treated with probiotics. This notification was supported by previous studies. 30 31 32 The significant decrease in BI after 30 days in patients treated with probiotics versus the patients treated without probiotics sheds light on the role of probiotic bacteria in decreasing the gingival inflammation due to its antimicrobial activity against pathogenic bacteria.

The inflammatory protease MMP-8 is released mainly by neutrophil and plays a role in pathogenesis of periodontal disease and transition from periodontal health to periodontal disease. 25 The GCF levels of MMP-8 were investigated in both examined groups at baseline and 30 days of periodontal therapy. The nonsurgical periodontal treatment of both studied groups revealed a significant decrease in GCF levels of MMP-8 in patients managed by nonsurgical periodontal treatment only, whereas a highly significant reduction in GCF levels of MMP-8 in patients managed by nonsurgical periodontal therapy with probiotic lozenges.

The significant decrease in GCF/MMP-8 levels after 30 days of initial periodontal therapy was consistent with some reports. 35 36 The previous researches documented a significant reduction in MMP-8 levels following nonsurgical periodontal therapy, which corresponded with our study. Furthermore, the highly significant decrease in GCF/MMP-8 in patients treated by nonsurgical periodontal treatment and probiotics was like the results of Szkaradkiewicz et al. 34 They reported that the application of periodontal therapy with tablets containing a strain of *L. reuteri* induced a significant reduction in GCF levels of proinflammatory cytokines, including TNF-α, interleukin-1β (IL-lβ), and IL-17, and improvement in periodontal clinical parameters (BI, PPD, CAL).

The clinical and immunological evaluations of probiotic lozenges containing *L. reuteri* as an adjunct for nonsurgical periodontal treatment in chronic periodontitis were studied by Ince et al. 31 They reported that the decrease in GCF/MMP-8 levels and increase in tissue inhibitor matrix metalloproteinase-1 (TIMP-1) levels were observed to be significant at 21 days ( *p* < 0.05), in both group of patients, those treated with SRP only and those with SRP and probiotics. These findings are in accordance with results of the present study. The authors of the previous study reported that the intergroup comparison of GCF/MMP-8 and TIMP-1 levels at 21 days of initial therapy was highly significant. This inspection is in conflict with results of this study that showed a nonsignificant reduction in GCF/MMP-8 between the examined groups at 30 days of periodontal treatment. For elucidation, this outcome may explain the need for studying a large population with long-term evaluation.

## Conclusion

The current study has suggested an improvement in periodontal parameters in both groups, especially in patients treated with probiotic lozenges. Moreover, there was a highly immunologic response by reduction in GCF/MMP-8 levels in patients managed by nonsurgical periodontal treatment and probiotic lozenges than in patients managed by nonsurgical periodontal treatment only. Further research on a large-scale population with a long duration of follow-up is needed to clarify the clinical, immunological, and microbiological responses to probiotics as an adjunctive to the nonsurgical periodontal therapy.
